# Bibliometric analysis of the top 100 cited articles in breast radiology

**DOI:** 10.1259/bjro.20230027

**Published:** 2023-05-23

**Authors:** Tsz Ki Ko, Denise Jia Yun Tan

**Affiliations:** 1 College of Life Sciences, Leicester Medical School, George Davies Centre, Leicester, UK; 2 Royal Wolverhampton Hospital Trust, Wolverhampton, UK

## Abstract

**Objective:**

Bibliometrics analysis is a widely used approach that enables influential research within specific fields to be identified

To identify the 100 most-cited articles in breast radiology and analyse the trend in breast imaging research.

**Methods and materials:**

A systematic search was conducted using the Thomson Rheuters Web of Science database. The results were ranked according to citation count and screened to create a single database. Data including first author, year of publication, journal, country of origin, primary institution, number of citations and average number of citations per year were extracted, as well as the impact factor and the 5-year impact factor of journals publishing the articles.

**Results:**

The systematic search yielded a total of 114,426 articles, after filters were applied to include papers that were available in English only. Citations for the 100 most-cited articles ranged from 515 to 3660. Half of the articles on the list were published between 2001 and 2010. Radiology has the most number of publications (*n* = 17), followed by JAMA-Journal of The American Medical Association (*n* = 9). CA-A Cancer Journal For Clinicians had the highest impact factor of 286.13. Mammogram (*n* = 49) was the most commonly studied modality, followed by Magnetic Resonance (*n* = 26). The most common topic of publication was diagnosis (*n* = 83).

**Conclusion:**

This research serves as a guide to the most influential articles on the topic of breast radiology.

## Introduction

Bibliometrics is a widely used approach that enables influential research within specific fields to be identified.^1^ It is based upon statistical methods, which allows the reviewer to evaluate published papers and assess core parameters such as research performance, impact, and the productivity of key authors in the field.^2^ In so doing, this style of research can be effective in identifying past trends within an area, and in enabling potential future directions to be identified in the process.^3^ Because evidence-based practice is such an underpinning force within the field of healthcare provision,^4^ the use of bibliometrics can therefore be both influential and impactful within the delivery of care to patients that is both safe and effective.^5^

The use of bibliometrics has a strong precedence in the field of radiology, with bibliometric analysis having been applied both in the field of radiology as a whole^6^ and within more specific subfields of the profession.^7^ The recent bibliometric analysis conducted by Oo and Chu,^8^ which focused on radiology of the head and neck was effective at producing a novel set of results that could have both clinical and research-related impact, and this work shall provide the framework for how this current study is conducted and reported. There is, to the best of this author’s knowledge, no bibliometric analysis that has been conducted on the issue of breast radiology, and so by providing a comprehensive assessment of this field, this current study shall seek to provide novel insights into this important area.

## Methods and materials

A bibliometric analysis of the most highly cited articles in breast radiology was carried out in September 2022. A search was conducted within Thomson Reuters Web of Science (WOS) using the following key terms:

- *Breast AND radiology OR*
- *Breast AND imaging OR*
- *Breast AND (X-ray OR radiograph) OR*
- *Breast AND mammogram OR*
- *Breast AND computed tomography OR*
- *Breast AND magnetic resonance OR*
- *Breast AND (ultrasound OR sonography) OR*
- *Breast AND interventional radiology OR*
- *Breast AND nuclear imaging OR*
- *Breast AND nuclear medicine*

This process returned a total of 145,396 articles. Filters were then applied to include full manuscripts that were available in English only (*n* = 114,426). The results were then sorted by the number of citations from the most-cited to the least-cited. This method was developed by Paladugu et.al^9^ and was used in several other studies. Each article was manually screened for inclusion. Articles were included if they focused on diagnostic imaging, interpretation, imaging technique, utility and role of different imaging modalities or trends in breast radiology. Articles that focused on benign breast pathologies other than breast cancer were also included. Articles are excluded if they are not related to breast radiology, or if the primary focus was not on the breast. Articles that focus primarily on therapeutic radiology were excluded.

**Figure 1.**
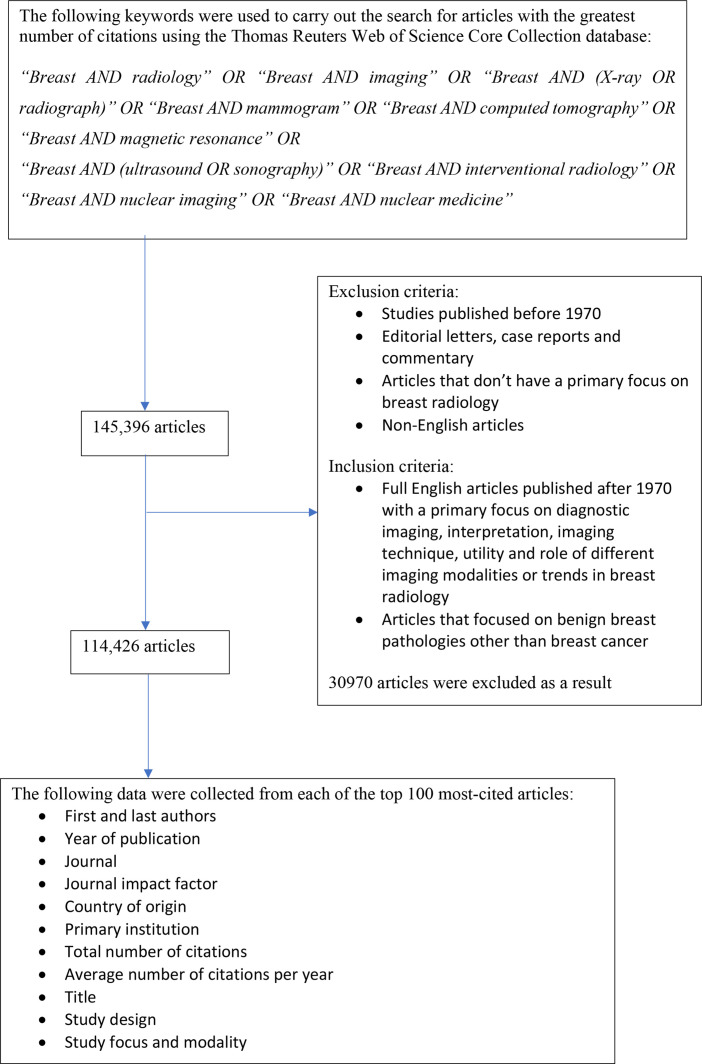
Flowchart depicting the selection of the top 100 most-cited articles on breast radiology

Data such as WOS citations, year published, first author, last author, primary institution, country of origin, journal, journal impact factor, title, study design, study focus and modality were collected for each article and assembled into a single database (As shown in Figure 1).

## Results

The top 100 most-cited articles has a mean citation number of 899.41 and a median of 772, as shown in Table 1. The number of citations ranged from 515 to 3660.

**Table 1.** The top 100 most-cited articles in breast radiology ranked in descending number of citations

Rank	Citations	Average citations per year	First author	Publication year
1	3660	159.13	Rueckert, D	1999
2	2783	89.77	OPHIR, J	1991
3	1796	112.25	Xu, MH	2006
4	1740	102.35	Berry, DA	2005
5	1719	114.6	Saslow, D	2007
6	1701	94.5	Bercoff, J	2004
7	1551	41.92	TABAR, L	1985
8	1516	101.07	Boyd, NF	2007
9	1417	109	Anwar R. Padhani*	2009
10	1373	91.53	Koh, DM	2007
11	1368	72	Chlebowski, RT	2003
12	1334	121.27	Beard, P	2011
13	1325	57.61	Young, H	1999
14	1295	53.96	Krouskop, TA	1998
15	1288	92	Mettler, FA	2008
16	1238	45.85	STAVROS, AT	1995
17	1210	71.18	Pisano, ED	2005
18	1175	65.28	Kriege, M	2004
19	1171	73.19	Itoh, A	2006
20	1122	56.1	Kolb, TM	2002
21	1098	84.46	Calonge, N	2009
22	1070	89.17	Welch, HG	2010
23	1068	71.2	Soret, M	2007
24	1043	69.53	Einstein, AJ	2007
25	1040	80	Montaldo, G	2009
26	988	164.67	Siu, AL	2016
27	959	41.7	Kuhl, CK	1999
28	958	53.22	Berg, WA	2004
29	956	68.29	Berg, WA	2008
30	917	131	Oeffinger, KC	2015
31	917	61.13	Doi, K	2007
32	916	114.5	Plana, JC	2014
33	898	52.82	Kennedy, JE	2005
34	890	89	Marmot, MG	2012
35	886	88.6	Bleyer, A	2012
36	877	32.48	BOYD, NF	1995
37	864	43.2	Nightingale, K	2002
38	853	60.93	Willmann, JK	2008
39	843	49.59	Chen, J	2005
40	840	38.18	Phelps, ME	2000
41	835	75.91	DeSantis, C	2011
42	835	64.23	Nelson, HD	2009
43	834	41.7	Humphrey, LL	2002
44	834	30.89	KERLIKOWSKE, K	1995
45	821	45.61	Warner, E	2004
46	815	26.29	STRAUSS, LG	1991
47	795	37.86	Manduca, A	2001
48	790	39.5	Nystrom, L	2002
49	787	87.44	Bamber, J	2013
50	772	45.41	Leach, MO	2005
51	772	40.63	Carney, PA	2003
52	766	30.64	Garra, BS	1997
53	758	30.32	Schwartz, LM	1997
54	757	44.53	Kuhl, CK	2005
55	757	31.54	Elmore, G	1998
56	755	26.03	NYSTROM, L	1993
57	752	53.71	Fletcher, JW	2008
58	740	30.83	Sipkins, DA	1998
59	711	64.64	Bardhan, R	2011
60	710	29.58	Powles, T	1998
61	693	31.5	Mandelson, MT	2000
62	689	76.56	Cosgrove, D	2013
63	687	76.33	Skaane, P	2013
64	684	25.33	BYRNE, C	1995
65	681	68.1	Berg, WA	2012
66	670	134	Sigrist, RMS	2017
67	667	30.32	Ntziachristos, V	2000
68	667	22.23	BIRD, RE	1992
69	649	324.5	McKinney, SM	2020
70	631	21.76	FLETCHER, SW	1993
71	629	41.93	Lehman, CD	2007
72	623	24.92	Niklason, LT	1997
73	622	32.74	Swan, J	2003
74	620	18.79	KAISER, WA	1989
75	596	25.91	Ophir, J	1999
76	590	49.17	Sardanelli, F	2010
77	588	24.5	Boyd, NF	1998
78	586	27.9	Freer, TW	2001
79	581	20.75	PARKER, SH	1994
80	578	28.9	Fear, EC	2002
81	576	64	Ciatto, S	2013
82	575	19.17	Miller, AB	1992
83	571	31.72	Rohren, EM	2004
84	568	56.8	Berg, WA	2012
85	567	40.5	Houssami, N	2008
86	563	35.19	Shankar, LK	2006
87	556	25.27	Gotzsche, PC	2000
88	553	23.04	Borgstein, P	1998
89	550	27.5	Guo, Y	2002
90	549	68.63	Friedewald, SM	2014
91	549	24.95	Tromberg, BJ	2000
92	543	38.79	Tanter, M	2008
93	542	45.17	Dijkers, EC	2010
94	538	25.62	Orel, SG	2001
95	536	28.21	Smith, RA	2003
96	533	66.63	Angelo, M	2014
97	528	24	Meaney, PM	2000
98	522	34.8	Kuhl, C	2007
99	517	30.41	Elmore, JG	2005
100	515	42.92	Lee, CH	2010

### Citations per year

Citations per year ranged from 13.92 to 1830 with a mean of 84.74 and a median of 45.40 per year. The article by Rueckert et al^10^ , titled ‘Nonrigid registration using free-form deformations: Application to breast MR images, is the most-cited article in the list and has the highest number of citations per year

### Year of publication

**Figure 2.**
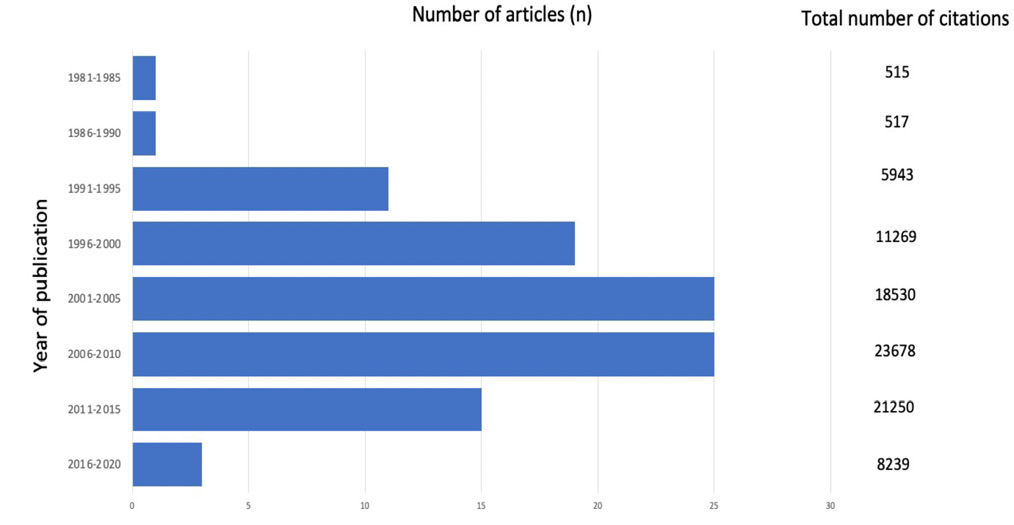
Number of articles across time

The articles were published between 1985 and 2020. 2007 had the greatest number of publications, with eight articles, followed by the year 2005 with seven articles. Half of the articles on the list were published between 2001 and 2010 (Figure 2).

### Most common first author

There was a total of 92 first authors on the top 100 list. Amongst them, Berg has the highest number of articles, with four articles. There were five authors who were first authors of more than one article to the top 100 list.

### Journals

The top 100 articles were published across 41 journals (Table 2). Radiology has the greatest number of publications (*n* = 17), followed by JAMA-Journal of The American Medical Association (*n* = 9). CA-A Cancer Journal For Clinicians had the highest impact factor of 286.13, the highest 5 year impact factor of 334.26 and the highest journal citation factor of 76.09, and it contributed to three articles on the list.

**Table 2.** Journals in which the 100 top-cited articles in breast radiology were published

Journal	**Number of articles**	Impact Factor (2021)	5 year impact factor	Journal citation factor (2021)	Total number of citations
RADIOLOGY	17	29.146	17.483	5.52	1,3465
JAMA-JOURNAL OF THE AMERICAN MEDICAL ASSOCIATION	9	157.335	101.13	10.32	7686
NEW ENGLAND JOURNAL OF MEDICINE	7	176.079	125.115	22.26	7913
LANCET	7	202.731	130.838	21.81	6024
ANNALS OF INTERNAL MEDICINE	6	51.598	38.361	6.01	5285
JOURNAL OF THE NATIONAL CANCER INSTITUTE	5	11.816	13.993	2.28	3955
JOURNAL OF NUCLEAR MEDICINE	4	11.082	9.837	2.59	3198
CA-A CANCER JOURNAL FOR CLINICIANS	3	286.13	334.259	76.09	3090
PROCEEDINGS OF THE NATIONAL ACADEMY OF SCIENCES OF THE UNITED STATES OF AMERICA	2	12.779	13.45	2.61	1507
ULTRASONIC IMAGING	2	2	1.93	0.5	4078
IEEE TRANSACTIONS ON ULTRASONICS FERROELECTRICS AND FREQUENCY CONTROL	2	3.267	3.287	1.01	2731
NEOPLASIA	2	6.218	8.247	1.09	1966
EUROPEAN JOURNAL OF CANCER	2	10.002	9.433	1.72	1915
ULTRASOUND IN MEDICINE AND BIOLOGY	2	3.694	3.36	0.92	1407
ULTRASCHALL IN DER MEDIZIN	2	5.445	5.853	1.58	1476
JOURNAL OF CLINICAL ONCOLOGY	2	50.717	38.801	5.62	1324
NATURE MEDICINE	2	87.241	68.31	12.6	1273
IEEE TRANSACTIONS ON MEDICAL IMAGING	1	11.037	12.369	2.54	3660
REVIEW OF SCIENTIFIC INSTRUMENTS	1	1.843	1.751	0.4	1796
AMERICAN JOURNAL OF ROENTGENOLOGY	1	6.582	5.201	1.52	1373
INTERFACE FOCUS	1	4.661	5.338	1.09	1334
COMPUTERIZED MEDICAL IMAGING AND GRAPHICS	1	7.422	6.141	1.4	917
JOURNAL OF THE AMERICAN SOCIETY OF ECHOCARDIOGRAPHY	1	7.722	8.357	1.28	916
NATURE REVIEWS CANCER	1	69.8	78.98	7.95	898
NATURE REVIEWS DRUG DISCOVERY	1	112.288	98.74	9.68	853
NANO LETTERS	1	12.262	12.709	1.95	843
MEDICAL IMAGE ANALYSIS	1	13.828	14.934	2.48	795
ACCOUNTS OF CHEMICAL RESEARCH	1	24.466	24.462	1.38	711
THERANOSTICS	1	11.6	12.201	2.52	670
NATURE	1	69.504	63.58	10.88	649
CANCER	1	6.921	7.802	1.22	622
CANCER EPIDEMIOLOGY BIOMARKERS & PREVENTION	1	4.09	5.594	0.98	588
IEEE TRANSACTIONS ON BIOMEDICAL ENGINEERING	1	4.756	5.674	1.16	578
LANCET ONCOLOGY	1	54.433	49.204	8.48	576
CANADIAN MEDICAL ASSOCIATION JOURNAL	1	16.859	14.14	2.04	575
JOURNAL OF THE AMERICAN COLLEGE OF SURGEONS	1	6.532	6.163	2.51	553
JOURNAL OF MAGNETIC RESONANCE IMAGING	1	5.119	4.801	1.21	550
CLINICAL PHARMACOLOGY & THERAPEUTICS	1	6.903	7.038	1.65	542
IEEE TRANSACTIONS ON MICROWAVE THEORY AND TECHNIQUES	1	4.381	4.348	1.12	528
JOURNAL OF THE AMERICAN COLLEGE OF RADIOLOGY	1	6.24	5.947	1.29	515
PROCEEDINGS OF THE INSTITUTION OF MECHANICAL ENGINEERS PART H-JOURNAL OF ENGINEERING IN MEDICINE	1	1.763	1.871	0.33	596

### Country and institution of origin

**Figure 3.**
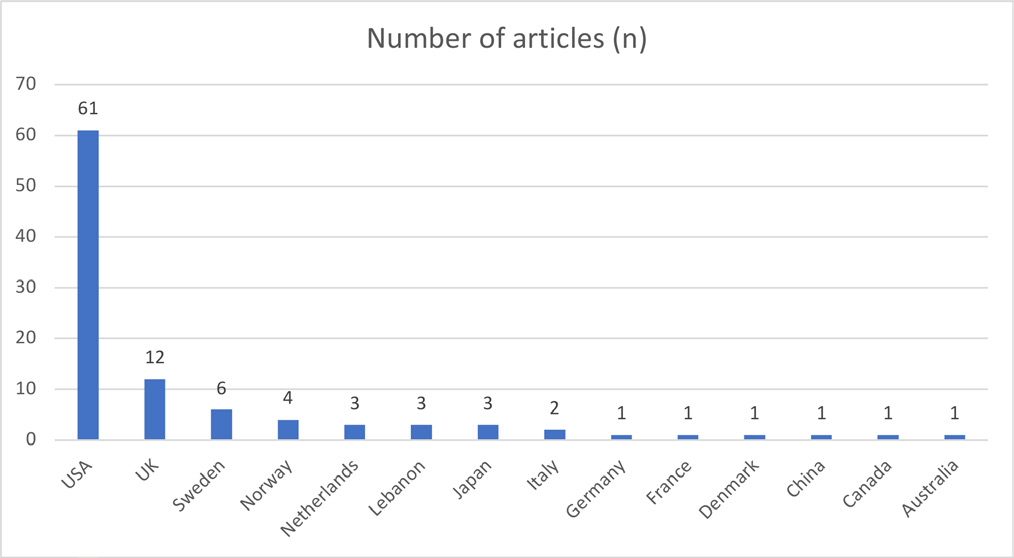
Country of the first author for 100 top-cited articles in breast radiology

The United States (USA) has the greatest number of publications on the list (*n* = 61), followed by the United Kingdom (UK) (*n* = 12). The results are displayed in Figure 3. In terms of the affiliated academic institutions of the first authors on the list, Stanford University and the University of Washington both have the highest number of articles, with four each.

### Study design

The majority of the study designs was prospective (*n* = 60), followed by reviews (*n* = 28), retrospective (*n* = 10) and a mix of prospective and retrospective (*n* = 2) (Table 3)

**Table 3.** Study designs of included papers

	Descriptor	Frequency (n)
Study design	Prospective	60
	Review	28
	Retrospective	10
	Mixed	2
Modality (15 articles focused on more than one modality)	Mammogram	49
	MR	26
	Ultrasound	26
	PET	10
	CT	5
	Others	5
Primary topic/ focus	Diagnosis	83
	Prognosis	1
	Treatment response	3
	Management	2
	Others	11
Related to breast cancer	Yes	90
	No	10

### Topic

Imaging modality, primary topic of the article and if the article was breast cancer-related were also evaluated for all of the articles on the list—83 of them focused on the diagnosis of breast pathology and 90 papers are related to breast cancer (Table 3)

## Discussion

This bibliometric analysis offers a range of interesting insights into the field of breast radiology. These findings include, as presented above, insights into how this field has developed over the years, and what the core focuses have been over time.

From the analysis conducted as part of this current study, the most-cited article in this field was the work of Rueckert and colleagues, which was published in the journal IEEE Transactionson Medical Imaging in 1999. This article has, at the time of conducting this analysis, received *n* = 3660 total citations. This, averaged across the 23 years since publication equates to approximately *n* = 159 citations being made of this paper every year; clearly indicating therefore that Rueckert and colleagues’ work continues to have a lasting and influential impact on the field of breast radiology. It should be noted that this paper, as with the second most-cited article in this field—that of Ophir et al^11^—was published before the turn of the century. Because of the way that bibliometric analysis works, this can tend to bias earlier published work towards appearing more influential.^12^ However, whilst this caveat should be borne in mind, the scale of influence that these papers had is incontrovertible.

The top three journals within this current analysis—Radiology; JAMA-Journal of the American Medical Association; and the New England Journal of Medicine—are not all journals which focus specifically on the field of radiology; indeed, only Radiology, with a total of *n* = 17 articles within this bibliometric analysis is focussed on this topic. This is a finding of interest because this does not support the findings of Oo and Chu,^8^ who noted that in their analysis of head and neck radiology, the top three journals were all radiology-centric in nature. The reasons for the disparity between the findings of Oo and Chu^8^ and those of this current bibliometric analysis may be the subject matter, with the issue of breast cancer, in particular, being considered more feasible or of interest to a wider, more general audience.

Next to be discussed is the journal impact factor of the journals within the top 100 list presented above. The impact factor of a journal is a blunt, yet influential approximation of the esteem and influence of that journal,^13^ which is calculated by dividing the citations of papers in that journal across the period of a year by the number of articles published in the previous 2 years.^13^ The higher the impact factor is, the more influential that journal is considered to be,^14^ and it is notable that the impact factor of the above, top three journals ranges considerably. For example, the impact factor for the journal Radiology is 29.1, whilst the second-ranked journal of JAMA-Journal of the American Medical Association (impact factor = 157.3) and third-ranked journal of New England Journal of Medicine (impact factor = 176.1) are far more influential. This again may indicate why breast radiology researchers targeted these journals for publishing their studies, due to the greater reach and impact of such journals.

It is also notable that, regarding the location of study, the 83% of the included papers were published by American or British research teams, with the vast majority of these*—n* = 61—being based in America. This is indicative of the research culture across clinical teams in these countries, and the resources available to clinical and research teams to develop the evidence base. This finding is highly conducive with those of other bibliometric analyses published in the field of radiology.^15^

From the analysis conducted as part of this current study, it is clear that the core focus in the field of breast radiology is breast cancer, with 90 papers within the selected 100 addressing this issue. This can potentially be related to the emotive nature of breast cancer^16^ and the impact that this awareness has on research funding decisions and opportunities.^17^ Whilst this is important in one sense—clearly it can be argued that the advances made in understanding breast cancer screening, identification and treatment can be attributed to the huge research resources provided to this area^18^—it is possible that this focus has limited advances in other areas of breast radiology. It is however notable that the volume of studies included in this bibliometric analysis peaked between 2001 and 2015, which may either indicate a shift in focus of research teams in recent years, or alternatively may be indicative of the primacy biases that are inherent within bibliometric analysis studies.^12^

Whilst nearly half of studies focussed on a primary modality of mammograms, this is indicative of practice in this field, although it should be noted that other core approaches such as PET or CT scans tend to be underrepresented within the top 100-cited articles in this area, and more perhaps needs to be done to address this imbalance. It is also notable that *n* = 60 of these studies were prospectively designed; this is important to address because unlike in retrospectively designed work, such studies are able to limit the potential of biases and confounding variables to influence findings.^19^ The rigour of these selected works is further underlined by the relatively high volume of review studies (*n* = 28). Well-conducted reviews can take into account important issues such as risk of bias within primary studies,^20^ and as such are considered to be near the apex of the evidence-based pyramid within healthcare research.^21^

As far as this author is aware, this is the first bibliometric analysis to have focused on the issue of breast radiology. This study has therefore produced a set of novel results that can provide researchers, radiologists, and other allied health professionals with information about the most impactful. Influential papers within this field, which in turn may inform both their approach to practice, and potential directions for future research projects. However, whilst this work is therefore likely to be influential in the above ways, it should be noted that there are a number of limitations associated with this study. It is important that these limitations are discussed in order for there to be a transparent discussion of the implications from this work. For example, it is possible that the search terms used incorporated some form of researcher bias into the search process.^8^ It is also of note that articles which focussed on therapeutic radiology were excluded from consideration for this analysis, which may have biased the inclusion of papers within this work; a future bibliometric analysis into therapeutic radiology may help redress this imbalance. Finally, this bibliometric analysis utilised output from WOS, but as noted in the past bibliometric analysis,^8^ citation data can differ considerably across core databases such as WOS, Google Scholar, PubMed or Scopus. However, whilst these limitations should be held in mind, it should also be noted that a central bias of this methodology—that towards earlier published papers—was addressed via the use of a citations per year metric, which is indicative of this work being rigorously conducted and transparent in nature.

In conclusion, this study has provided a detailed and comprehensive overview of the top 100 most-cited papers in the field of breast radiology. In so doing, this study can provide core insights for both clinicians and researchers alike, and lead them to understand the most influential papers within this important field. It is anticipated that this may positively impact on the way that this area is addressed in the future, and that the evidence base in this field can be further advanced as a result.
